# Impact of an *SLC30A8* loss-of-function variant on the pancreatic distribution of zinc and manganese: laser ablation-ICP-MS and positron emission tomography studies in mice

**DOI:** 10.3389/fendo.2023.1171933

**Published:** 2023-06-16

**Authors:** George Firth, Eleni Georgiadou, Alexander Griffiths, Maral Amrahli, Jana Kim, Zilin Yu, Ming Hu, Theodora J. Stewart, Isabelle Leclerc, Haruka Okamoto, Daniel Gomez, Philip J. Blower, Guy A. Rutter

**Affiliations:** ^1^ School of Biomedical Engineering and Imaging Sciences, King’s College London, St Thomas’ Hospital, London, United Kingdom; ^2^ Section of Cell Biology and Functional Genomics, Division of Diabetes, Endocrinology and Metabolism, Imperial Centre for Translational and Experimental Medicine, Imperial College London, London, United Kingdom; ^3^ London Metallomics Facility, King’s College London, London, United Kingdom; ^4^ Centre hospitalier de l’Université de Montréal (CHUM) Research Center and Faculty of Medicine, University of Montreal, Montreal, QC, Canada; ^5^ Regeneron Pharmaceuticals, Inc., Tarrytown, NY, United States; ^6^ Lee Kong Chian School of Medicine, Nanyang Technological, University, Singapore, Singapore

**Keywords:** SLC30A8, diabetes, pancreas, zinc, manganese, LA-ICP-MS, positron emission tomography

## Abstract

**Introduction:**

Common variants in the *SLC30A8* gene, encoding the secretory granule zinc transporter ZnT8 (expressed largely in pancreatic islet alpha and beta cells), are associated with altered risk of type 2 diabetes. Unexpectedly, rare loss-of-function (LoF) variants in the gene, described in heterozygous individuals only, are protective against the disease, even though knockout of the homologous *SLC30A8* gene in mice leads to unchanged or impaired glucose tolerance. Here, we aimed to determine how one or two copies of the mutant R138X allele in the mouse *SLC30A8* gene impacts the homeostasis of zinc at a whole-body (using non-invasive ^62^Zn PET imaging to assess the acute dynamics of zinc handling) and tissue/cell level [using laser ablation inductively coupled plasma mass spectrometry (LA-ICP-MS) to map the long-term distribution of zinc and manganese in the pancreas].

**Methods:**

Following intravenous administration of [^62^Zn]Zn-citrate (~7 MBq, 150 μl) in wild-type (WT), heterozygous (R138X^+/−^), and homozygous (R138X^+/+^) mutant mice (14–15 weeks old, *n* = 4 per genotype), zinc dynamics were measured over 60 min using PET. Histological, islet hormone immunohistochemistry, and elemental analysis with LA-ICP-MS (Zn, Mn, P) were performed on sequential pancreas sections. Bulk Zn and Mn concentration in the pancreas was determined by solution ICP-MS.

**Results:**

Our findings reveal that whereas uptake into organs, assessed using PET imaging of ^62^Zn, is largely unaffected by the R138X variant, mice homozygous of the mutant allele show a substantial lowering (to 40% of WT) of total islet zinc, as anticipated. In contrast, mice heterozygous for this allele, thus mimicking human carriers of LoF alleles, show markedly increased endocrine and exocrine zinc content (1.6-fold increase for both compared to WT), as measured by LA-ICP-MS. Both endocrine and exocrine manganese contents were also sharply increased in R138X^+/−^ mice, with smaller increases observed in R138X^+/+^ mice.

**Discussion:**

These data challenge the view that zinc depletion from the beta cell is the likely underlying driver for protection from type 2 diabetes development in carriers of LoF alleles. Instead, they suggest that heterozygous LoF may paradoxically increase pancreatic β-cell zinc and manganese content and impact the levels of these metals in the exocrine pancreas to improve insulin secretion.

## 1 Introduction

The expression and activity of zinc transporters—Zrt-/Irt-like protein (ZIP) transporters responsible for import of zinc into the cytosol and ZnT transporters mediating export from the cytosol—are tightly controlled in health but are dysregulated in diseases such as cancer and diabetes. ZnT8, encoded by *SLC30A8*, is a zinc transporter expressed almost exclusively in the islet cells of the endocrine pancreas. Its main role in β-cells is the transport of Zn^2+^ against a concentration gradient into insulin secretory granules for insulin crystallisation and storage (1). Common variants in *SLC30A8* have been identified through genome-wide association studies (GWAS) in humans and affect type 2 diabetes (T2D) risk (2, 3). Unexpectedly, given the importance of Zn^2+^ for insulin storage and secretion, haploinsufficiency of *SLC30A8* is protective against the development of T2D in humans. Thus, loss of function (LoF) mutations have been identified, which decrease the risk of developing T2D without adverse phenotypes (4, 5). One of the most common LoF variants, p.Arg138* (rs200185429, c.412C>T, also termed R138X), results in a premature stop codon and the formation of a truncated and unstable protein. Heterozygosity for R138X, which decreases the risk of developing T2D by 53%, is extremely rare in western Europe, with 0.02% of individuals possessing the allele, but more than 10-fold more common in western Finland (>0.2%) (4).

We previously developed a mouse model with the R138X human LoF variant expressed from the endogenous locus (“knock-in”) and demonstrated that Zn-depleted islets with no detectable ZnT8 protein are still capable of secreting insulin in response to hyperglycaemia. These mice maintain normal body weight, glucose tolerance, and β-cell mass (6). A human study in people with one R138X allele demonstrated that enhanced insulin secretory responsiveness to glucose, combined with enhanced proinsulin processing, links *SLC30A8* LoF mutations and protection from developing T2D (5).

The acute dynamic handling of zinc (particularly its delivery from blood to tissues and redistribution thereafter) and chronic accumulation and storage of zinc in these ZnT8 LoF variants remain poorly understood. Given that pancreatic zinc is highly concentrated in islet β-cells in healthy subjects and that homozygous R138X LoF mutations in ZnT8 result in zinc-depleted islets (as measured by dithizone staining) (6), our hypothesis was that trafficking and retention of zinc in the pancreas would be perturbed, resulting in a global reduction in pancreatic zinc. Recent developments in imaging technology are making testing of hypotheses such as this possible. We have previously shown the potential of the positron-emitting zinc radioisotope ^62^Zn for non-invasively monitoring zinc trafficking *in vivo* beyond 1 day with PET imaging (7, 8). Here, we describe the application of ^62^Zn to measure zinc dynamics, combined with inductively coupled plasma mass spectrometry (ICP-MS) and laser ablation-ICP-MS (LA-ICP-MS), which maps static metal quantity at cellular resolution. To our knowledge, this is the first time that PET and LA-ICP-MS and immunohistochemistry have been combined in this way. This combined approach provides evidence for an unexpected action of SLC30A8 variants on zinc and manganese distribution in the pancreas, with possible relevance for the understanding of altered T2D risk in heterozygous carriers of LoF alleles.

## 2 Materials and methods

### 2.1 Animals

All animal experiments were performed in accordance with the Animals (Scientific Procedures) Act, 1986, with protocols approved by the Animal Welfare and Ethical Review Body for King’s College London (KCL, St Thomas’ Campus) and by the Regeneron Pharmaceuticals Institutional Animal Care and Use Committee. The SLC30A8 R138X mouse lines were established as previously described (6) and maintained as part of a breeding colony at the Central Biological Services facility of the Hammersmith Campus, Imperial College London (UK Home Office Project Licence PPL PA03F7F07 to I.L.). In brief, R138X mice were generated initially from a C57BL/6NTac background using VelociGene technology, replacing nucleotide 409 from a cytosine to a thymidine in exon 3 causing a premature stop codon. Mice were housed (up to five mice per cage) in a controlled environment (12 h light/dark cycle, 22°C ± 1°C, 60%–70% humidity) and fed *ad libitum* with chow (Purina Laboratory 23 Rodent Diet 5001; LabDiet). Following genotyping using a protocol detailed previously (6), male WT and heterozygous and homozygous mice (30 ± 2 g, 14–15 weeks old) were transported to the Imaging Chemistry and Biology (ICAB) Department at KCL for PET imaging, ICP-MS, and histology studies.

### 2.2 Production of ^62^Zn for PET imaging

[^62^Zn]Zn-citrate was produced as previously described (7). In brief, this was achieved by proton irradiation (27 MeV, 35 µA for 9 h) of a copper foil target that was subsequently dissolved in concentrated hydrochloric acid and hydrogen peroxide. The dissolved ^62^Zn was purified using ion exchange cartridges, and the eluted [^62^Zn]Zn-citrate was diluted with 0.9% NaCl for *in vivo* studies to give a final citrate concentration of 8.5 mM and pH of 6–7.

### 2.3 PET imaging of zinc trafficking

Blinded PET imaging was carried out on 12 mice (14–15 weeks of age, *n* = 4 per genotype) on the same day that they were transferred from Imperial to KCL. Mice were anaesthetised by isoflurane inhalation (2%–2.5%, Animal care, York, UK, in O_2_) and the tail vein cannulated. Mice were then transferred to the scan bed in a prone position, and a bolus of [^62^Zn]Zn-citrate (~7 MBq, 150 μl, 8.5 mM trisodium citrate) was administered intravenously *via* the tail vein cannula. A dynamic PET scan was continuously acquired from 0 min post-injection (p.i.) for 1 h using a nanoScan-PET/CT (Mediso, Budapest, Hungary) in list mode with 400–600 keV energy window and coincidence relation of 1:3. A CT scan was then acquired for anatomical reference (55 keV X-ray, exposure time of 1,000 ms, 360 projections, and pitch 1). Animals were maintained under isoflurane anaesthesia at 37°C throughout the scan and vital signs were monitored.

PET projection data were processed with the Monte Carlo-based full-3D iterative algorithm reconstruction using the Tera-tomo^®^ software package (four iterations, six subsets; 0.4 mm isotropic voxel size) with attenuation, scatter, and dead-time corrections. Data were re-binned and reconstructed into a series of 1-min time frames for the first 5 min, 5-min time frames for the next 25 min, and then 10-min time frames for the remaining scan period. The data were then visualised and quantified using VivoQuant^©^ (InviCro, Boston, USA) software with regions of interest (ROIs) manually drawn over the brain, heart, liver, kidney, bladder, and thigh muscle. The CT images were used to define the boundaries of the organs. Time–activity curves (TACs) and maximum intensity projection (MIP) images were generated and expressed as percentage injected dose per gram of tissue (%ID/g). Areas under the curves (AUCs) for the regional TACs from 30 to 60 min (AUC_30–60 min_) were determined.

### 2.4 *Ex vivo* biodistribution

At the end of the previously described imaging protocol (approximately 75 min p.i.), mice were culled *via* cervical dislocation, and tissues were collected. All tissues were washed with phosphate-buffered saline (PBS, Sigma, 806552), blotted dry, weighed, and then counted using a gamma counter (1282 Compugamma; LKB, window set to channels 175–220 to measure 511 keV gamma rays following positron decay). *Ex vivo* biodistribution data were presented as %ID/g, where ID represents the sum of the activity of all body parts excluding the tail.

### 2.5 Sample preparation for zinc and manganese quantification

Approximately 50 mg of mouse pancreas tissue was transferred to 15 ml acid-cleaned trace metal grade HDPE centrifuge tube (VWR) and oven dried at 70°C overnight. After drying, samples were weighed and then digested overnight in 0.45 ml of Optima grade concentrated HNO_3_ (67%–69% w/w; Fisher Scientific) and 0.15 ml Optima grade concentrated H_2_O_2_ (30–32% w/w; Sigma Aldrich) at 60°C. Once the tissue had been completely digested, samples were diluted with purified water from a Milli-Q system (Merck Millipore) to a total volume of 13.5 ml. Samples were diluted by a factor of 50 to minimise matrix effects and were doped with an internal Ga standard (Teledyne Leeman Labs).

### 2.6 Quantification of pancreatic zinc and manganese by ICP-MS

The prepared samples were analysed at the London Metallomics Facility, KCL using a Perkin Elmer NexION 350D Inductively Coupled Plasma Quadrupole Mass Spectrometer (ICP-QMS) under Dynamic Reaction Cell (DRC) mode with a Cetac ASX-520 autosampler coupled to a SeaSpray glass nebuliser fitted to a quartz cyclonic spray chamber. Quantification was permitted through calibration standards, which were prepared from a 100 mg L^−1^ TraceCert multi-element standard (Sigma Aldrich, UK). Element concentrations of the calibrants were between 0.1 and 2,500 µg L^−1^, and all calibration solutions, like the samples, were doped with an internal Ga standard.

### 2.7 Tissue processing for histological and elemental distribution analysis

Pancreata (*n* = 2—3 per genotype) were harvested and fixed overnight in 4% formalin at 4°C. Fixed tissues were submitted to UCL IQPath (London, UK) where they were embedded in paraffin and sliced to provide sequential 5-μm sections for histological and elemental distribution analysis. Alternate sections were stained with haematoxylin and eosin (H+E) and imaged with a Hamamatsu Nanozoomer S630 digital slide scanner to visualise general pancreatic architecture. Adjacent sections were left unstained for LA-ICP-MS and immunohistochemical studies.

### 2.8 LA-ICP-MS imaging

Explanted pancreata tissue sections of 5-μm thickness from all genotypes were analysed for zinc and manganese distribution, with phosphorus signal acting as a cell density map. Areas for ablation were determined based on sequential sections stained with H+E to identify islet and exocrine tissue. Prior to LA-ICP-MS analysis, paraffin-embedded pancreas sections were dewaxed by 5-min washes in xylene (three times) and 70% EtOH (three times). For the LA-ICP-MS experiments, an Analyte Excite 193 nm ArF*excimer-based LA system (Teledyne Photon Machines, Bozeman, MT, USA), equipped with a HelEx II two-volume ablation cell (Teledyne CETAC Technologies, Omaha, USA) was used. The LA system was coupled to a Thermo Fisher Scientific iCAP TQ ICP-mass spectrometer (Thermo Fisher Scientific, Waltham, MA, USA) *via* the Aerosol Rapid Introduction System (ARIS). Tuning of the instrument settings was performed using a NIST SRM 612 glass certified reference material (National Institute for Standards and Technology, Gaithersburg, MD, USA) and optimising for low laser-induced elemental fractionation by monitoring of ^238^U^+^/^232^Th^+^ ratios, low oxide formation (<1%) *via* ^232^Th^16^O^+^/^232^Th^+^ ratios, and high sensitivity for ^59^Co^+^, ^115^In^+^, and ^238^U^+^. LA-ICP-MS images were acquired using a fixed dosage mode, with a vertical and horizontal spatial resolution of 3 and 10 µm, respectively (for acquisition parameters, see **Supplementary Table S1**). Glass slides were mounted inside a bespoke three-slide sample holder of the HelEx II two-volume ablation cell. To correct for instrumental drift, a series of NIST 612 standard ablation scans were performed before and after each pancreas section. ICP-MS and positional data were reconstructed using the HDF-based Image Processing software (HDIP, Teledyne Photon Machines Inc., Bozeman, MT, USA). A bespoke pipeline, written in Python (version 3.8), was used to generate elemental images from reconstructed data and statistics. Negative values, attributed to instrumental noise, were replaced with zeros. Image quantification was performed in HDIP by drawing ROIs around islets (identified by their low phosphorus signal) and surrounding exocrine tissue. Pixel intensities in counts were then averaged and compared between groups. Frequency distributions were also generated in GraphPad Prism using a bin width of 2.

### 2.9 Immunohistochemistry

Unstained pancreata sections were dewaxed by submersion in Histoclear (Sigma, UK) and then washed in decreasing concentrations of EtOH. Permeabilised pancreatic slices were blotted with ready-diluted anti-guinea pig insulin (Agilent Technologies, USA) and anti-mouse glucagon (Sigma, UK) primary antibodies (1:1,000). Slides were visualised by subsequent incubation with Alexa Fluor 488 (1:1,000) and 568-labelled donkey anti-guinea pig and anti-mouse antibodies (1:1,000). Samples were mounted on glass slides using Vectashield^®^ (Vector Laboratories, USA) containing 4′,6-diamidino-2-phenylindole (DAPI). Images were captured on a Zeiss Axio Observer Z1 motorised inverted widefield microscope fitted with a Hamamatsu Flash 4.0 Camera using a Plan-Apochromat 206/0.8 M27 air objective with Colibri.2 LED illumination. Data acquisition was controlled with Zeiss Zen Blue 2012 Software.

### 2.10 Insulin secretion *in vivo*

To measure glucose-stimulated changes in circulating insulin levels *in vivo*, two cohorts of male mice, each consisting of R138X heterozygous mice and their wild-type littermates, were analysed. One cohort (*n* = 11 R138X^+/-^; *n* = 4 R138X^+/+^) was 14 weeks of age and fed regular chow, whereas the other cohort (*n* = 6 R138X^+/-^; *n* = 8 R138X^+/+^) was 1 year of age and fed 60% high fat diet (HFD) (Research Diet Cat. No. D12492i) for 28 weeks. Mice were fasted overnight (16 h) followed by oral gavage of glucose (Sigma) at 2 g/kg body weight. Submandibular bleeds were performed at 0, 15, and 30 min post-injection for insulin measurements. Secreted insulin in plasma was measured using the Mouse Insulin ELISA (Mercodia, Uppsala, Sweden) or the High Range Mouse Insulin ELISA (ALPCO, Salem, NH).

### 2.11 Statistical analysis

Data were analysed using GraphPad Prism (v7.0–9.0) using appropriate tests (Bonferroni-corrected T-test or ANOVA, as indicated). *p*-values <0.05 were considered significant.

## 3 Results

### 3.1 Kinetic delivery of zinc from blood to tissues over 1 h remains largely unchanged in homozygous and heterozygous R138X mice compared to WT mice

**Figure 1:**
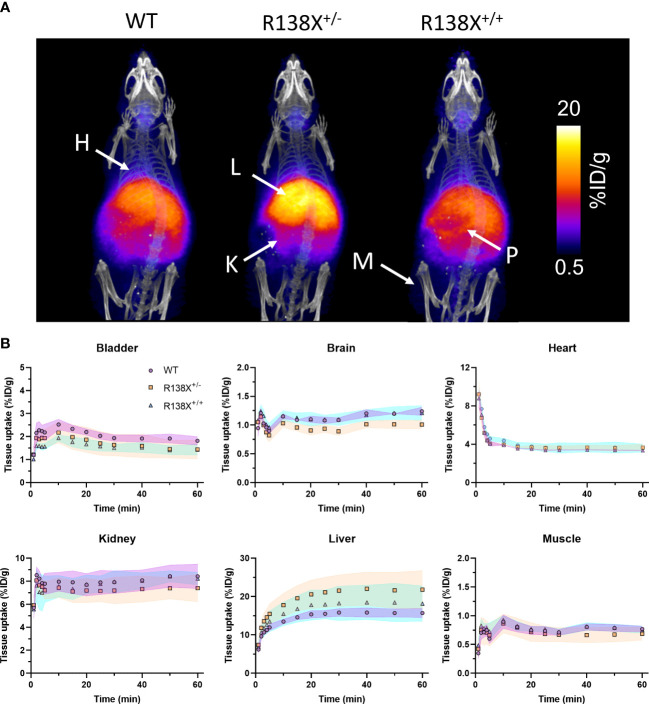
Zinc kinetics determined by PET imaging after intravenous administration of ^62^Zn in male R138X and WT mice. **(A)** Representative maximum intensity projection (MIP) PET/CT images of WT, R138X^+/−^, and R138X^+/+^ mice (14–15 weeks old) at 0–60 min p.i. following i.v. administration of [^62^Zn]Zn-citrate (H = heart, K = kidney, L = liver, P = pancreas and M = muscle). **(B)** Time activity curves (TACs) for WT (•), R138X^+/−^ (▪), and R138X^+/+^ (▴) mice in major organs of interest. Shaded areas represent one SD from the mean value (*n* = 4 mice per genotype).

Given that previous publications have shown that pancreatic islets lacking functional SLC30A8 are zinc depleted (6, 9), we first investigated whether zinc delivery to the whole pancreas is perturbed in mice possessing SLC30A8 LoF variants. PET allows the mapping of zinc trafficking across not just the pancreas but also the whole body simultaneously. Therefore, the impact of the R138X variant on global zinc trafficking was also studied. PET images revealed the typical biodistribution for ^62^Zn and ^63^Zn as seen in previous studies in mice and humans (7, 10, 11), including uptake in the heart, liver, kidneys, pancreas, intestines, and salivary glands (**Figure 1A**). PET quantification in all three groups revealed that ^62^Zn is rapidly cleared within minutes from the blood following i.v. injection. All comparisons of retention in tissues were performed by analysing the AUC between 30 and 60 min, excluding the first 30 min to avoid the complications arising from positron-emitting ^62^Cu present at the time of injection—an unavoidable consequence of the complex decay of ^62^Zn *via* ^62^Cu. By 15 min, heart uptake in all genotypes had reached a plateau of ~3.6%ID/g (**Figure 1B**), and AUC values between 30 and 60 min showed no significant difference between genotypes (**Supplementary Figure S1**). Kidney uptake plateaued 1 min after i.v. administration with no significant difference between groups. Bladder activity peaked at a modest 1.5%–2% ID/g for all groups. At 30–60 min p.i., bladder activity was somewhat lower in R138X^+/−^ (AUC; R138X^+/−^, 29.7 ± 4.2; WT, 37.8 ± 1.8; *p* = 0.0089; **Supplementary Figure S1**) and R138X^+/+^ mice (AUC; R138X^+/+^, 28.9 ± 2.1; WT, 37.8 ± 1.8; *p* = 0.0051; **Supplementary Figure S1**) compared to WT. Liver uptake peaked at approximately 30 min p.i. with R138X^+/−^ mice showing significantly higher accumulation thereafter compared to WT mice (AUC; 290.5 ± 31.3 vs. 210.0 ± 7.5 respectively, *p* = 0.0046; **Supplementary Figure 1**). R138X^+/+^ mice also demonstrated higher liver uptake at 30–60 min p.i. compared to WT mice (AUC; 245.2 ± 31.8 vs. 210.0 ± 7.5, respectively; not significant, *p* = 0.1919; **Supplementary Figure S1**) but less than heterozygotes (AUC; 245.2 ± 31.8 vs. 290.5 ± 31.3, respectively, *p* = 0.084; **Supplementary Figure S1**). Brain uptake was low but detectable, with mice heterozygous for R138X showing slightly lower ^62^Zn retention at 30–60 min p.i. compared to WT and R138X^+/+^ mice (AUC; R138X^+/−^, 13.5 ± 0.5; WT, 16.1 ± 0.3; R138X^+/+^, 16.0 ± 0.8; *p* < 0.003; **Supplementary Figure S1**).

**Figure 2:**
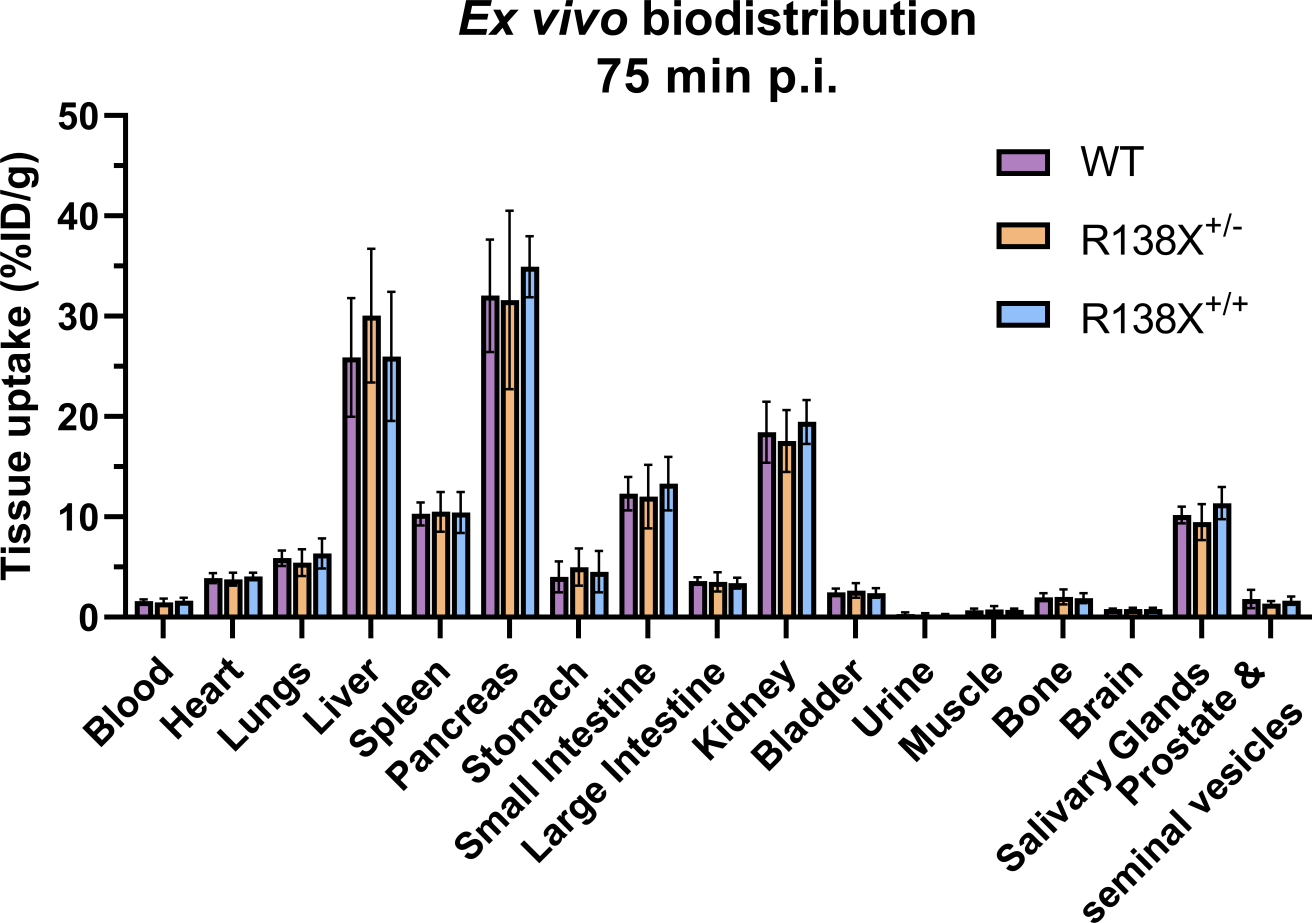
*Ex vivo* biodistribution of [^62^Zn]Zn-citrate in male R138X and WT mice at 75 min p.i. *Ex vivo* biodistribution analysis was determined at 75 min p.i. in male mice (*n* = 5–6 per genotype) by measuring radioactivity in excised organs by gamma counting and normalising to weight to give %ID/g. Graphs represent mean ± SD.

Visualising the pancreas with ^62^Zn-PET is challenging due to the amorphous nature of the gland, inadequate CT contrast, and background ^62^Zn uptake into surrounding abdominal organs. However, *ex vivo* radioactivity measurements permit comparisons to be made. Therefore, following PET imaging, mice were culled, and tissues were excised for radioactivity measurement. There was a 15-min delay between the end of the PET scan and the time of culling due to time dedicated to CT acquisition; therefore, small changes in zinc retention could occur during this time leading to differences between PET quantification and *ex vivo* data. ^62^Zn uptake at 75 min p.i. was marginally (not significantly) higher in R138X^+/+^ pancreata (34.9 ± 3.0 vs. 32.1 ± 5.6% ID/g, respectively, *n* = 5–6, *p* = 0.3335; **Figure 2**) and R138X^+/−^ liver (30.1 ± 6.7 vs. 25.9 ± 5.9% ID/g, respectively, *n* = 5–6, *p* = 0.5106; **Figure 2**) compared to the WT organs. Although small but statistically significant differences were observed in AUC data, these did not translate into significant differences in uptake in *ex vivo* biodistribution data.

### 3.2 Zinc and manganese distribution in endocrine and exocrine pancreas is significantly altered in R138X compared to WT mice

**Figure 3:**
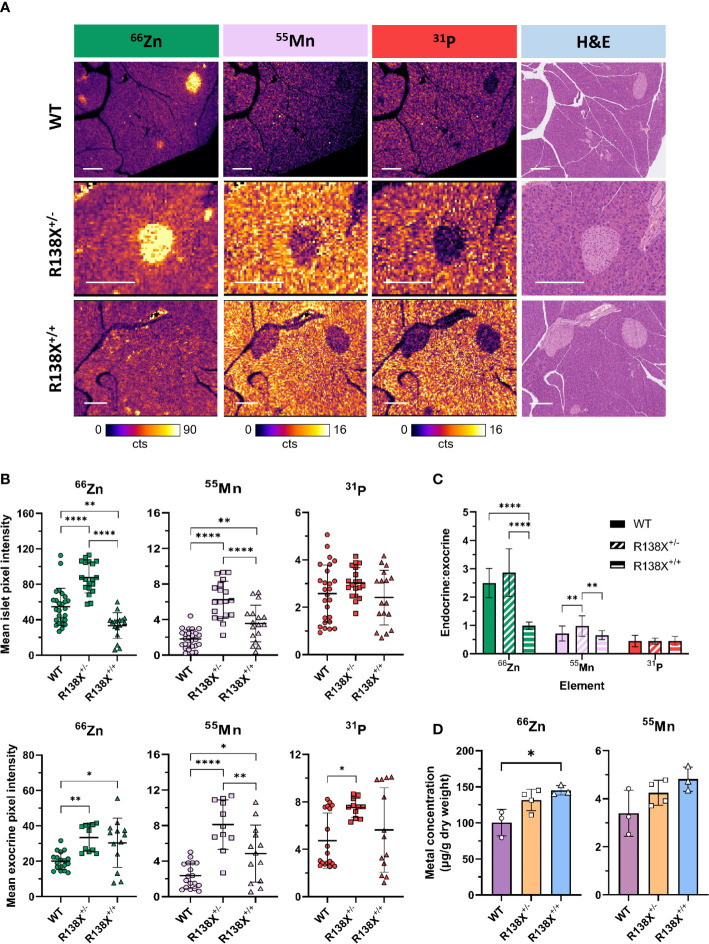
Metal distribution and quantification in pancreata from R138X and WT mice. **(A)** Representative LA-ICP-MS images for ^66^Zn, ^55^Mn, and ^31^P obtained from the same 5-μm pancreas section from age-matched 14–15-week-old R138X and WT mice. Pancreatic islets have high zinc levels in WT and R138X^+/−^ mice compared to surrounding exocrine tissue, but R138X^+/+^ mice lacking functioning ZnT8 have a uniform distribution of zinc across both endocrine and exocrine compartments. Scale bar is 250 μm. **(B)** Mean pixel intensity from ROI quantification of LA-ICP-MS images for endocrine (top) and exocrine (bottom) tissue. Scatter plots represent individual islets (*n* = 19–27 per genotype) and exocrine tissue (*n* = 11–18) from separate pancreas sections from two to three male animals per genotype, analysed in parallel on the same day. **(C)** Endocrine to exocrine ratio for ^66^Zn, ^55^Mn, and ^31^P derived from ROI quantification of LA-ICP-MS images. **(D)** Total zinc and manganese content in WT and R138X mouse pancreata (*n* = 3–4) measured by ICP-MS. Data were analysed for significance using a one-way ANOVA with Tukey’s *post-hoc* test for multiple comparisons *p < 0.05; **p < 0.01; ****p < 0.0001.

To confirm that R138X islets are zinc depleted, as previously reported (6), we next used LA-ICP-MS, which is a more sensitive technique than dithizone staining and offers a higher resolution than PET and thus permits the investigation of the distribution of elements across a tissue section. LA-ICP-MS revealed the expected zinc distribution in WT mouse pancreas; significantly higher concentrations of zinc were found in islets compared to the surrounding exocrine tissue (see **Figure 3A**), by a factor (endocrine to exocrine ratio) of 2.49 ± 0.52 by ROI quantification. Zinc content was reduced by 38.7% in R138X^+/+^ islets compared to WT islets (33.4 ± 14.5 counts, *n* = 17 vs. 54.5 ± 20.8 counts, *n* = 26; *p* = 0.0013; **Figure 3B**). In contrast, exocrine pancreatic zinc was significantly higher in R138X^+/+^ (30.4 ± 13.9 counts, *n* = 13) than in WT islets (20.1 ± 5.0 counts, *n* = 18; *p* = 0.0104; **Figure 3B**) but remained similar to that in heterozygote carriers (R138X^+/−^, 33.4 ± 7.7 counts, *n* = 11; *p* = 0.7126; **Figure 3B**). Overall, homozygous R138X^+/+^ mice displayed a homogenous distribution of zinc across the entire pancreas, in stark contrast to WT (endocrine:exocrine ratio of 0.99 ± 0.13, *n* = 21 vs. 2.49 ± 0.52, *n* = 27, respectively; *p* < 0.0001; **Figure 3C**) and R138X^+/−^ (endocrine:exocrine ratio of 0.99 ± 0.13, *n* = 21 vs. 2.86 ± 0.84, *n* = 19, respectively; *p* < 0.0001; **Figure 3C**). Compared to WT, R138X^+/−^ mice displayed 1.61-fold higher islet zinc (R138X^+/−^: 87.6 ± 17.4 counts, *n* = 19; WT: 54.5 ± 20.8 counts, *n* = 26; *p* < 0.0001; **Figure 3B**) and 1.66-fold higher exocrine zinc (R138X^+/−^: 33.4 ± 7.7 counts, *n* = 11; WT: 20.1 ± 5.0 counts, *n* = 18; *p* = 0.0015; **Figure 3B**). Consequently, endocrine to exocrine zinc ratio was also higher in R138X^+/−^ mice compared to the ratio in WT mice (2.86 ± 0.84, *n* = 19 vs. 2.49 ± 0.52, *n* = 27 respectively; *p* = 0.0774; **Figure 3C**).

Frequency distributions were generated from the pixel intensities for zinc from the ROIs (**Supplementary Figure S5**). For endocrine tissue, homozygous carriers for R138X showed two major populations—one narrow low-intensity peak that was left-shifted compared to WT and a second broader peak that was similar to WT. Mice heterozygous for R138X displayed a broad peak with the majority of endocrine pixel intensities higher than WT. For exocrine tissue, both R138X genotypes showed peak distributions, which were broader and shifted right with a higher pixel intensity compared to WT.

Given the importance of several transporters, such as ZIP14 and ZnT10, that were initially identified as zinc transporters but have since also been implicated in manganese homeostasis (12, 13), we examined whether the R138X variant in *SLC30A8* impacted manganese handling in the pancreas. In contrast to zinc in WT mice, manganese was found at substantially greater levels in the exocrine tissue than in endocrine tissue, with an endocrine to exocrine concentration ratio of 0.72 ± 0.26. This ratio was significantly higher in R138X^+/−^ mice than in WT (0.98 ± 0.36, *n* = 19 vs. 0.72 ± 0.26, *n* = 27, respectively; *p* = 0.0049; **Figure 3C**) but remained similar in R138X^+/+^ mice (0.72 ± 0.26, *n* =27 vs. 0.66 ± 0.16, *n* = 21; *p* = 0.7405; **Figure 3C**). Manganese levels were significantly higher in R138X^+/+^ islets compared to WT islets (3.56 ± 2.05 counts, *n* = 17 vs. 1.81 ± 1.03 counts, *n* = 26, respectively; *p* = 0.0047; **Figure 3B**) and were also higher in R138X^+/+^ exocrine tissue compared to WT exocrine tissue (4.85 ± 3.21 counts, *n* = 13 vs. 2.36 ± 1.43, *n* = 18, respectively; *p* = 0.0225; **Figure 3B**). Manganese content in both R138X^+/+^ islet and exocrine tissue was lower than in those tissues of R138X^+/−^ mice, which displayed the highest values across both pancreatic tissue compartments (islet, WT: 3.56 ± 2.05 counts, *n* = 17, R138X^+/−^: 6.22 ± 2.10 counts, *n* = 19; *p* < 0.0001; **Figure 3B**; and 4.85 ± 3.21 counts, *n* = 13 vs. 8.11 ± 2.79 counts, *n* = 11; *p* = 0.0069; **Figure 3B**, respectively). Manganese levels were 3.0- and 3.4-fold higher in the islet and exocrine tissue, respectively, in R138X^+/−^ mice compared to WT mice (6.22 ± 2.10 counts, *n* = 19 vs. 1.81 ± 1.03 counts, *n* = 26; *p* < 0.0001; **Figure 3B**; and 8.11 ± 2.79 counts, *n* = 11 vs. 2.36 ± 1.43 counts, *n* = 18; *p* < 0.0001; **Figure 3B**, respectively).

Phosphorus, used here as a control and as a general morphological indicator, followed a similar trend to manganese with more phosphorus present in the exocrine than endocrine tissue for all groups. Thus, for WT tissue, an endocrine to exocrine ratio of 0.45 ± 0.20 was calculated, which remained constant across other genotypes (R138X^+/−^: 0.45 ± 0.11, *n* = 21; R138X^+/−^: 0.46 ± 0.16, *n* = 19; *p* > 0.98; **Figure 3C**). Phosphorus was only significantly changed in R138X^+/−^ exocrine tissue, where levels were 1.6-fold higher than in WT (7.50 ± 0.80 counts, *n* = 11 vs. 4.73 ± 2.33 counts, *n* = 18, respectively; *p* = 0.0182; **Figure 3B**).

### 3.3 R138X mice have altered total pancreatic zinc and manganese compared to WT mice

We then investigated whether altered distribution of zinc and manganese in R138X mice affects total pancreatic metal levels. Bulk metal analysis by ICP-MS revealed a 1.4-fold increase in zinc in R138X^+/+^ pancreas compared to WT (145.3 ± 6.6 vs. 100.3 ± 18.4 μg/g dry weight, respectively, *p* = 0.015; **Figure 3D**) and a trend toward an increase (1.3-fold) in R138X^+/−^ pancreas compared to WT (131.7 ± 14.9 vs. 100.3 ± 18.4 μg/g dry weight, respectively, *p* = 0.0547; **Figure 3D**). Although manganese showed a similar trend as zinc—R138X^+/+^ mice showing the highest levels, followed by R138X^+/-^ and then WT—differences were not significant. Together with LA-ICP-MS, these results indicate a shift in zinc and manganese handling in the pancreas of R138X carriers.

### 3.4 Zinc levels are heterogeneous across individual pancreatic islet cells

**Figure 4:**
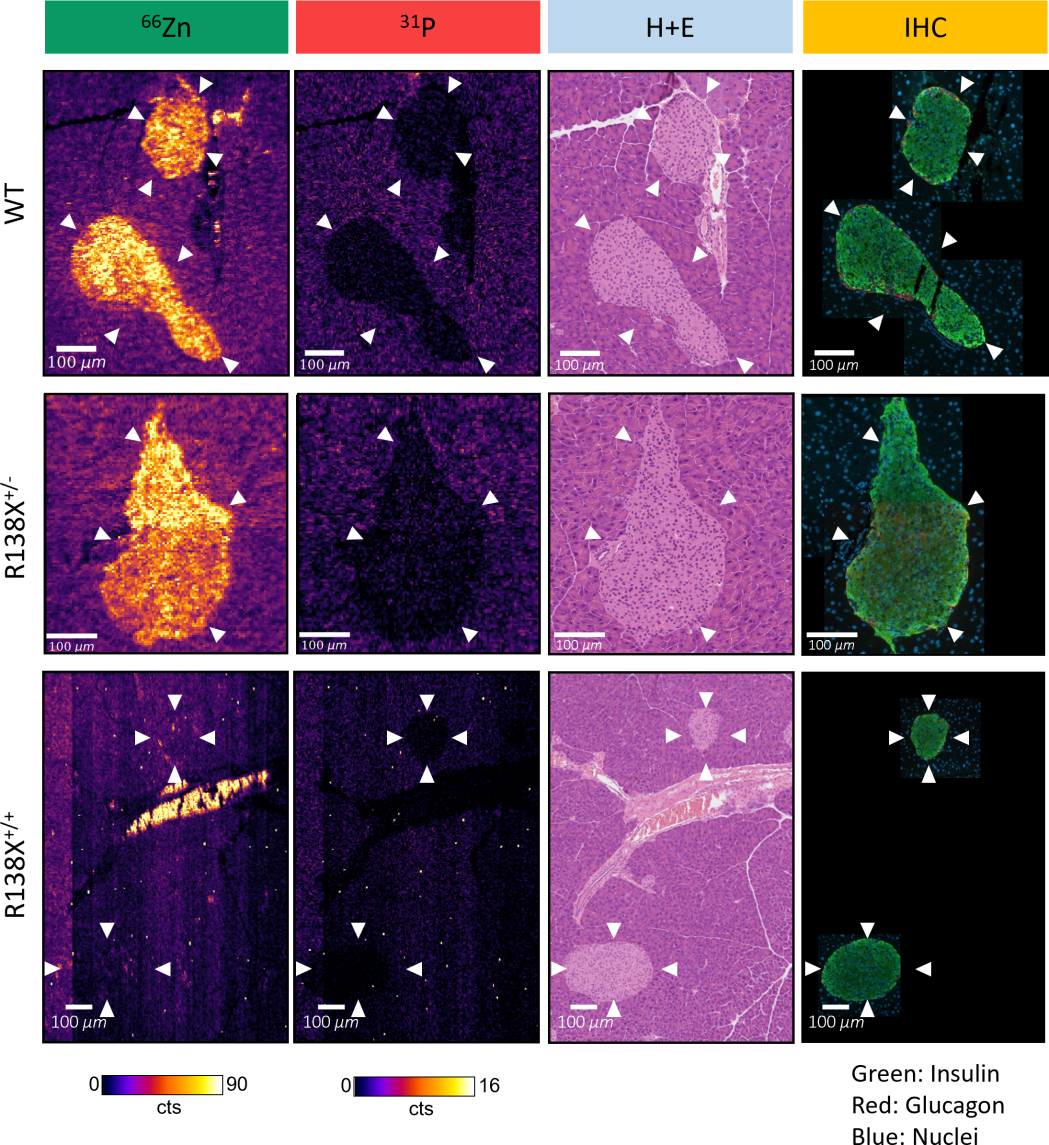
High-resolution LA-ICP-MS reveals heterogeneity of zinc within R138X and WT pancreatic islets. Representative ^66^Zn and ^31^P LA-ICP-MS images of selected islets (indicated with arrowheads) from each genotype are displayed alongside haematoxylin and eosin (H+E) stain and pancreatic hormone immunohistochemistry (IHC) from sequential tissue sections. The high zinc content structure in the centre of the R138X^+/+^ LA-ICP-MS image is a blood vessel, with the signal likely corresponding to individual red blood cells which were identified from a sequential H+E section.

High-resolution LA-ICP-MS images, with a spatial resolution of 3 μm, were generated from one mouse per genotype to visualise the heterogeneity in zinc content within individual islets (**Figure 4**). General observations remained the same as the 10-μm images—islets were zinc-rich in WT and R138X^+/−^ mice and relatively zinc-depleted in R138X^+/+^ mice. However, an improvement in spatial resolution revealed heterogeneity in the zinc content of all islets. In WT and R138X^+/−^ islets, the zinc content was vastly different between cells, with some possessing levels similar to exocrine cells and others ~10-fold higher. Although the mean pixel intensities for zinc in the endocrine and exocrine pancreas were close to uniform in R138X^+/+^ mice based on the 10-μm images, some regions of higher zinc content were observed in the 3-μm images and were mostly localised to the distal region of the islet shown in **Figure 4**. Immunohistochemistry (IHC) images from a sequential slide with insulin and glucagon antibodies, used to stain β- and α-cells, respectively, were generated to identify the cell types associated with high zinc. Representative merged IHC images for each genotype are shown in **Figure 4** and are separated into red (glucagon positive cells), green (insulin positive cells), and blue (nuclei) channels in **Supplementary Figure S6**. All islets, regardless of genotype, showed the typical core–mantle arrangement of β- and α-cells. Homozygous R138X mice possessed insulin-positive islets (**Supplementary Figure S6**) without functional ZnT8, as shown previously by Kleiner et al. (6)

### 3.5 Heterozygous ZnT8 R138^+/−^ mice show a tendency towards enhanced glucose-stimulated changes in circulating insulin *in vivo*

The results above demonstrated clear differences in metal content between wild-type and hetero- and homozygous R138X mice. In previous studies, we have reported increased glucose-induced insulin secretion in homozygous R138X^+/+^ versus wild-type animals, when both were maintained on a high fat diet (5). We extended these studies to heterozygous R138X^+/−^ mice in the present study and observed a similar tendency towards augmented secretion in the mutant mice, which was most marked for animals on high fat diet (**Supplementary Figure S7**).

## 4 Discussion

### 4.1 Altered zinc and manganese distribution in the pancreas may contribute to altered T2D risk in SLC30A8 variant carriers

ZnT8 has attracted much attention because of its link to T2D (2, 4, 5), but its importance in the control of glucose homeostasis remains a matter of debate. Whilst several studies in mice have reported that complete inactivation of ZnT8 has a detrimental impact on pancreatic β-cell function, others have suggested no effect on, or even improvement in, function (14). In studies of the mechanisms by which altered ZnT8 activity influences T2D risk, the primary focus thus far has been on characterising metabolic phenotype—notably glucose-regulated insulin secretion—and there has been little investigation of the impact of LoF mutations on zinc homeostasis.

It is known that islets from mice carrying rare ZnT8 LoF alleles have normal insulin content, but that zinc content is considerably reduced (by >70%, based on dithizone staining or the use of the fluorescent zinc sensor zinquin in ZnT8 knockout mice) (6), in line with studies in which SLC30A8 is eliminated completely (3, 9, 15). Together, these results suggest that normal insulin crystallisation is not required to maintain normal insulin content. However, the effect, if any, of ZnT8 LoF mutations on acute zinc kinetics across the whole body or on the metal content across the endocrine and exocrine pancreas—and how these effects may in turn impact β-cell function—have not been previously studied.

In the present work, we examined the acute (in response to injection of positron-emitting zinc) and chronic (assessing endogenous levels) handling of zinc in wild-type mice and in an R138X mouse model that mimics the human *SLC30A8* LoF mutation (p.Arg138*) associated with lowered T2D risk (4–6). PET imaging with a radioisotope of zinc, ^62^Zn, allowed us to measure the dynamics of zinc delivery *in vivo* and revealed only very minor differences between genotypes in whole-body zinc trafficking. Despite previous studies showing that R138X mice have zinc-depleted islets (6), ^62^Zn uptake into the pancreas at 75 min post-injection was not significantly different between genotypes. This suggests that ZnT8 does not play a role in the acute uptake of zinc into the pancreas within the time frame studied. Elemental maps generated by LA-ICP-MS, however, revealed drastic changes in the distribution of endogenous zinc between endocrine and exocrine components of the pancreas (well beyond the millimetre-scale resolution of PET) in R138X mice. Whilst in WT mice, islets could be readily identified in LA-ICP-MS images by their zinc content alone, in mice homozygous for R138X, they could not. Instead, zinc was more uniformly distributed throughout the pancreas. Importantly, both zinc and manganese contents were increased in the islets of R138X^+/−^ mice versus wild-type or R138X^+/+^ animals. Importantly, our studies provide conformation using ICP-MS of earlier demonstrations using dithizone of lower islet zinc content in mice null for ZnT8 (3) or carrying LoF alleles (6). Whilst the above reported data only on homozygous animals, in one study proving data on dithizone staining in ZnT8^+/−^ islets, a tendency was apparent for increased zinc levels (16).

Using zinc-binding magnetic resonance contrast agents (17–19), or PET probes (20), to perform imaging during glucose stimulated insulin secretion *in vivo* would be of significant value to confirm a reduction in zinc secretion from islets in R138X mice. In the case of heterozygous R138X carriers, zinc content was increased in both endocrine and exocrine components of the pancreas (based on ROI quantification of LA-ICP-MS images) compared to WT mice, resulting in an overall increase in pancreas zinc concentration (confirmed by ICP-MS). Combining these findings with the ^62^Zn biodistribution data, which showed no significant change in acute zinc uptake in pancreas, our data reveal that the increase in pancreatic zinc content in R138X mice is not acute (minutes to hours) but instead takes place over an extended period.

A recent study in humans concluded that heterozygous carriers of the R138X LoF allele had increased insulin secretion capacity and a lower risk of developing T2D in the absence of adverse effects (5). Unfortunately, the same studies were not possible in human carriers homozygous for R138X due to the extreme rarity of these variants. Our findings suggest that an increase, rather than decrease—as previously assumed—in levels of zinc or manganese in rare LoF carriers may drive improved β-cell performance or survival, and insulin output, lowering T2D risk. It is also conceivable that altered cross-talk between the exocrine and endocrine pancreatic compartments contributes to improved insulin production in these individuals (21).

The mechanisms through which deletion of a single allele of ZnT8 may result in elevated islet (presumed to be largely beta cell) zinc content remain unclear. One likely possibility is that this is the result of compensatory mechanisms, which may include the upregulation of the remaining *Slc30a8* allele, or changes in the expression or function (at the mRNA or protein levels) of other zinc transporters or importers, and the numerous zinc binding proteins that control total cellular and organellar zinc content. In any case, an association between increased zinc levels and elevated secretory function is more in line with comparisons between WT and ZnT8 KO mice (3, 9). The molecular mechanisms linking elevated zinc and improved secretion also remain unclear.

### 4.2 Zinc distribution among cell types is heterogeneous within individual islets

Given that several different cell types make up pancreatic islets, the heterogeneity in the zinc signal observed in all genotypes with LA-ICP-MS suggests that each cell type has different requirements for zinc and that zinc content could be heterogenous even within the same cell type. Although this heterogeneity was visible in LA-ICP-MS images with a spatial resolution of 10 μm, it was not sufficient to delineate individual cells within the islet. With the instrumentation used here, the more elements measured, the lower the overall resolution, and so by concentrating on measuring solely zinc, we were able to push the LA-ICP-MS instrument to generate images with a spatial resolution of 3 μm (bearing in mind that other studies typically ablate a full pancreas section with a spatial resolution of >50 μm) (22). The increase in resolution comes with major drawbacks—less sample ablated means less signal and an increase in scanning time translates to a significant increase in cost. Given that we were investigating the potential for delineating zinc content of individual cell types within the pancreatic islet, high-resolution LA-ICP-MS images were generated from selected islets from one mouse per genotype. These images still lacked the necessary resolution to perform this, but they permitted us to observe that zinc concentration is heterogenous across the islet. Several endocrine cell types (notably β, α, δ, ε, and PP) make up the typical architecture of pancreatic islets, but it is the β-cells that are renowned for their high intra-granular zinc concentrations (estimated at 30 mM in insulin secretory granules of healthy mice (23)). The LA-ICP-MS and IHC techniques described here provided the means to assess quantification of zinc in individual cell populations. Although the zinc content of R138X^+/+^ islets was significantly reduced compared to WT, regions of higher zinc content were observed and were mostly localised to the distal region of the islet. It is known that α-cells also express ZnT8 and so are likely to be effected in a similar manner to β-cells in R138X^+/+^ mice (9, 24). Although it is possible that these regions of higher zinc content are associated with other islet cell populations such as δ or PP cells, the latter are scarce in adult rodent islets (<5% of total endocrine cells) (25). Instead, our data indicate that considerable heterogeneity in zinc levels exists within the β-cell population (60%–80% of all islet endocrine cells) (25), consistent with the functional and molecular heterogeneity of these cells (26–32). Alterations in the degree of heterogeneity, or in the connectivity between discrete β-cell populations (33, 34), may thus impact islet function after deletion of functional ZnT8 alleles. A recent study on human-derived beta-like cells carrying the LoF variant demonstrated that cytoplasmic-free zinc content (based on the FRET zinc sensor eCALWY-4) remains the same in homozygous and heterozygous for R138X than controls (35). A significant reduction in the transport of zinc into insulin granules will likely increase the availability of cytosolic zinc in LoF variants of ZnT8.

The expected consequence of β-cells no longer having high concentrations of zinc, in the case of ZnT8 LoF mutants, is severe disruption to the normal synthesis, storage, and release of insulin. In fact, as reported by Kleiner et al., the effects on insulin processing and secretion and *in vivo* glycemia are small (6) consistent with earlier data on ZnT8 knockout mice (3, 9). The proposed mechanism for this unexpected finding is that the majority of insulin is no longer stored in its hexameric form and that, in response to hyperglycaemia, can be released more rapidly as insulin is already in a soluble form. Nevertheless, earlier studies, chiefly performed on homozygous ZnT8 KO mice [reviewed in (14)] demonstrate poorer glucose homeostasis versus wild-type littermates, especially on diabetes-prone backgrounds (e.g., human islet amyloid polypeptide, IAPP-transgenic mice) (16), or after high fat feeding (36). These findings suggest that preserved zinc content is required to maintain normal β-cell health and insulin secretion in the longer term, especially under circumstances that may more closely mimic human conditions (i.e., westernised diets and metabolic stress).

Despite R138X^+/+^ mice having zinc-depleted islets, bulk zinc concentration in the pancreas was the highest of all genotypes as measured by ICP-MS. LA-ICP-MS quantification suggested that regional differences, notably an increase in exocrine zinc, are responsible for this. Given that most of the pancreas is comprised of exocrine tissues (>95%), small changes in the zinc content of the exocrine tissue will have a large effect on the bulk metal content as determined by ICP-MS; conversely, changes in zinc levels in islets may not significantly affect total pancreatic zinc. Nevertheless, this finding may point to a role for islet cell zinc uptake as a means of maintaining lower zinc levels in neighbouring acinar cells, an effect disrupted by inheritance of LoF *SLC30A8* alleles.

An unexpected finding during our studies was that the R138X mutation also disrupts the normal homeostasis of manganese in the pancreas. Total pancreatic manganese trended higher in R138X mice compared to WT based on ICP-MS analysis, and manganese in the endocrine and exocrine pancreas was increased in both heterozygous and homozygous R138X mice compared to WT controls. These data contrast with previous *in vitro* results where haploinsufficiency of ZnT8 led to reduced intracellular zinc content, but not manganese, in Min-6 cells (37). Further studies are required to determine whether the differences observed are due to a direct or indirect effect. For example, does an upregulation of manganese transporters, such as ZIP14, lead to an increase in manganese accumulation in pancreatic cells as a compensatory mechanism for LoF mutations in ZnT8? Measurements of transporters such as ZIP14, ZIP8, and ZnT10 would help determine whether their altered expression/activity is responsible for increased pancreatic manganese, although are not feasible in human subjects. Whilst no substantive changes in the expression of the transcripts encoding these transporters were reported after homozygous deletion of ZnT8 in the mouse (3, 9), or in homozygous R138X mice (6), changes in heterozygous mice are conceivable at both the mRNA and protein level. In any case, a further important possibility flowing from the present studies is that alterations in the metal ion (zinc and manganese) content, and hence the function or viability of cells within the exocrine compartment of the pancreas, may ultimately feedback on pancreatic endocrine cells, impacting the secretion of insulin and other islet hormones. Consistent with this possibility, exocrine–endocrine cross-talk has recently been shown to be detrimental to β-cells in an inherited form of diabetes, Maturity-onset diabetes of the young-8 (MODY8) (21).

Following the principle established here of imaging metal trafficking with radionuclides, the positron emitting radionuclide ^52^Mn could be used to track the acute trafficking of manganese over a period of 4 weeks using the same mouse model (38). ^52^Mn has previously been shown to localise to the pancreas with elevated concentrations compared to the blood and other tissues. It is retained in the pancreas at least 13 days post-injection and may provide a tool to monitor β-cell function (39). The findings described in this paper suggest that the majority of manganese is actually found in the exocrine tissue, and others have shown using LA-ICP-MS that only with large concentrations of exogenous manganese (used as a magnetic resonance imaging contrast agent) does manganese move from exocrine to endocrine cells after 24 h (40). Autoradiography of pancreas sections at several time points following intravenous administration of ^62^Zn and ^52^Mn would allow for the major acute delivery compartment to be determined.

### 4.3 Limitations of the study

The findings reported here are based on the use of relatively low numbers of animals, which were all male, reflecting the low throughput and high cost of the complex imaging and analytical techniques deployed. Parallel examination of the metabolic phenotype, e.g., glucose tolerance tests, were not available for all three genotypes, although studies on wild-type and homozygous mice from the same colony (not shown) revealed the absence of evident differences in glucose tolerance for animals maintained on regular chow diet, in line with published studies (6). Finally, the current report is limited to work in rodent models, with extrapolation to humans subject to uncertainty.

## 5 Conclusions

In summary, our data demonstrate that mice bearing the human *SLC30A8* R138X loss-of-function mutation exhibit drastic differences in metal distribution within the pancreas compared to WT littermates. Further studies will be required to see how changes in acute and chronic handling of metals impacts pancreatic function and ultimately its influence on the mechanisms that govern T2D risk. The multi-faceted, multi-scale approach described here provides an example of what is now possible using recent advancements in metallomics technology.

## Data availability statement

The raw data supporting the conclusions of this article will be made available by the authors, without undue reservation.

## Ethics statement

The animal study was reviewed and approved by Animal Welfare and Ethical Review Body for King’s College London and by the Regeneron Pharmaceuticals Institutional Animal Care and Use Committee.

## Author contributions

All authors provided contributions to study conception and design, acquisition of data or analysis and interpretation of data, drafting the article, and final approval of the version to be published. Data were collected by GF, EG, AG, MA, MH, HO, and DG. Analysis was carried out by GF. All authors contributed to the article and approved the submitted version.
