# Supplementary material for: Impact of an SLC30A8 loss-of-function variant on the pancreatic distribution of zinc and manganese: laser ablation-ICP-MS and positron emission tomography studies in mice
Source: Front Endocrinol (Lausanne). 2023 Jun 16;14:1171933. doi: 10.3389/fendo.2023.1171933 (PMC10313231; doi:10.3389/fendo.2023.1171933)
Supplement: Supplementary file 1 [file DataSheet_1.pdf]

frontiers

Supplementary Material

Impact of an *SLC30A8* loss-of-function variant on the pancreatic distribution of zinc and manganese: laser ablation-ICP-MS and positron emission tomography studies in mice

George Firth,[1*†] Eleni Georgiadou,[2] Alexander Griffiths,[3] Maral Amrahli,[3] Jana Kim,[1] Zilin Yu,[1] Ming Hu,[2] Theodora J. Stewart,[3] Isabelle Leclerc,[2,6] Haruka Okamoto,[4] Daniel Gomez,[4] Philip J. Blower[1] and Guy A. Rutter.[2,5,6*†]

[1]School of Biomedical Engineering & Imaging Sciences, King's College London, St Thomas' Hospital, London, SE1 7EH, UK.

[2]Section of Cell Biology and Functional Genomics, Division of Diabetes, Endocrinology and Metabolism, Imperial Centre for Translational and Experimental Medicine, Imperial College London, London W12 0NN, UK.

[3]London Metallomics Facility, King's College London, 4th Floor Franklin-Wilkins Building, London, SE1 9NH, UK.

[4]Regeneron Pharmaceuticals, Inc., Tarrytown, NY 10591.

[5]Lee Kong Chian School of Medicine, Nanyang Technological, University, Singapore, Republic of Singapore.

[6]CHUM Research Center and Faculty of Medicine, University of Montreal, Quebec, H2X 0A9, Canada.

*** Correspondence:**
Corresponding Authors
george.firth@kcl.ac.uk, g.rutter@imperial.ac.uk, guy.rutter@umontreal.ca

[†]Joint corresponding authors

Keywords: SLC30A8, diabetes, pancreas, zinc, manganese, LA-ICP-MS, positron emission tomography.

Table 1. Experimental parameters and data acquisition parameters used for LA-ICP-MS imaging.

	**10 µm LA-ICP-MS**	**4 µm LA-ICP-MS**	**NIST 612 Scans**
**Teledyne Photon Machines Analyte Excite**			
**Energy density (J cm^2)**	0.81	0.81	2.02
**Repetition rate (Hz)**	333	375	375
**Scan speed (µm s^{-1})**	333	100	1332/1000
**Beam waist diameter (µm)**	10 (circle)	3 (circle)	40 (circle)
**Scanning mode**	Fixed Dosage	Fixed Dosage	Fixed Dosage
**Scanning direction**	Uni-directional	Uni-directional	Uni-directional
**Effective dosage (shots per position)**	10	15	10/15
**He Carrier gas flow rate (L min^{-1})**	0.5	0.5	0.5
**Thermo Fisher Scientific iCAP TQ ICP-mass spectrometer**			
**RF power (W)**	1550	1550	1550
**Ar plasma gas flow rate (L min -1)**	14	14	14
**Ar auxiliary gas flow rate (L min -1)**	0.8	0.8	0.8
**Nebuliser gas flow rate (L min -1)**	1.03	1.03	1.03
**He gas flow rate (L min-1)**	3.25	3.25	3.25
**Acquired m/z ratios (amu)**	^{31}P, ^{55}Mn, ^{66}Zn	^{31}P, ^{66}Zn	^{31}P, ^{55}Mn, ^{66}Zn
**Respective dwell times (ms)**	2, 3.8, 19.4	10.4, 27.2	2, 3.8, 19.4<br>10.4, 27.2
**Total scan cycle time (ms)**	29	40	29/40

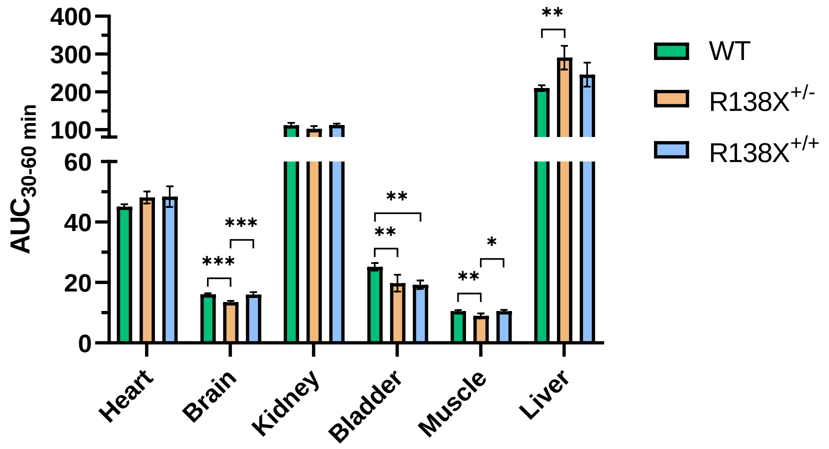

Figure S1 R138X mice demonstrate minor differences in zinc kinetics compared to WT mice by ^{62}Zn-PET quantification. AUC from 30 to 60 min p.i. were calculated for each subject (n = 4 mice per genotype) and analysed for significance using a one-way Anova with Tukey's *post-hoc* test for multiple comparisons, *, $p < 0.05$; **, $p < 0.01$; ***, $p < 0.001$.

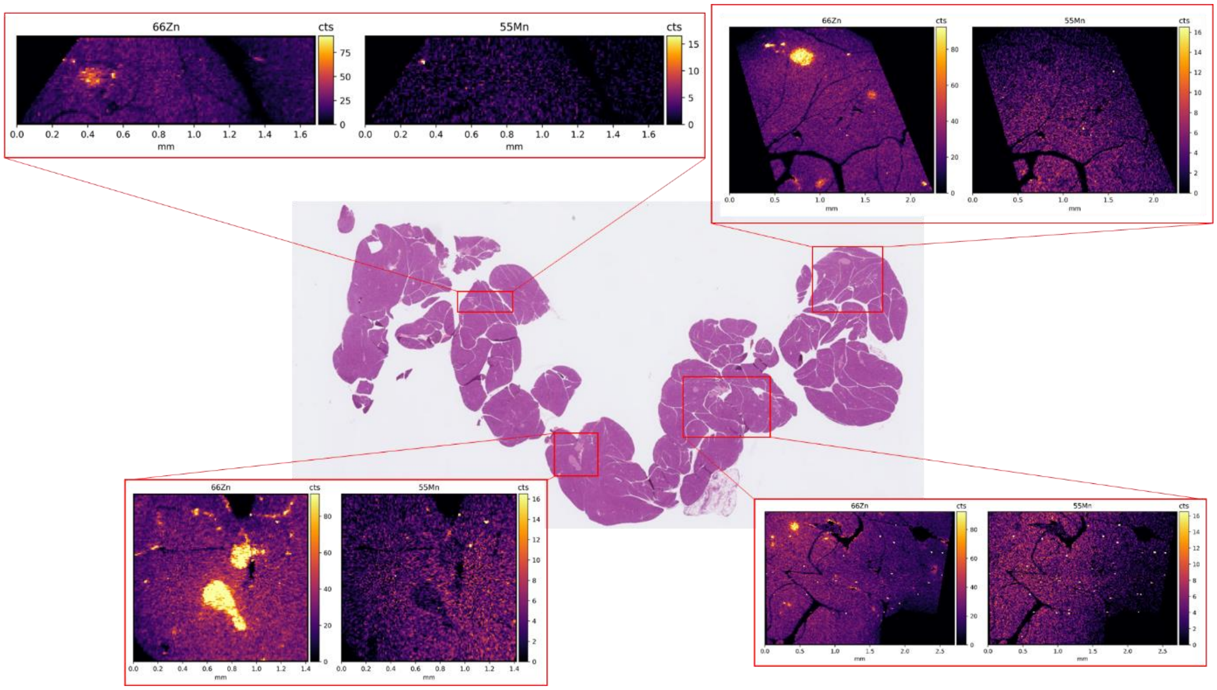

Figure S2 LA-ICP-MS of a pancreas from a wild-type mouse. ^{66}Zn and ^{55}Mn images are shown with ablated regions highlighted on a haemotoxylin and eosin-stained pancreas to visualise exocrine and islet tissue.

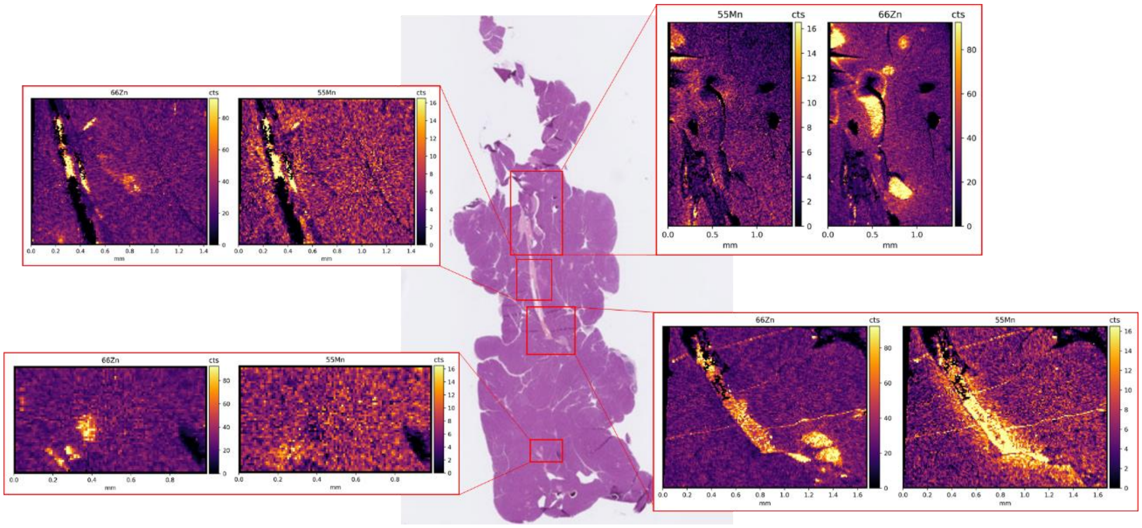

Figure S3 **LA-ICP-MS of a pancreas from a heterozygous R138X mouse.** ^{66}Zn and ^{55}Mn images are shown with ablated regions highlighted on a haemotoxylin and eosin-stained pancreas to visualise exocrine and islet tissue.

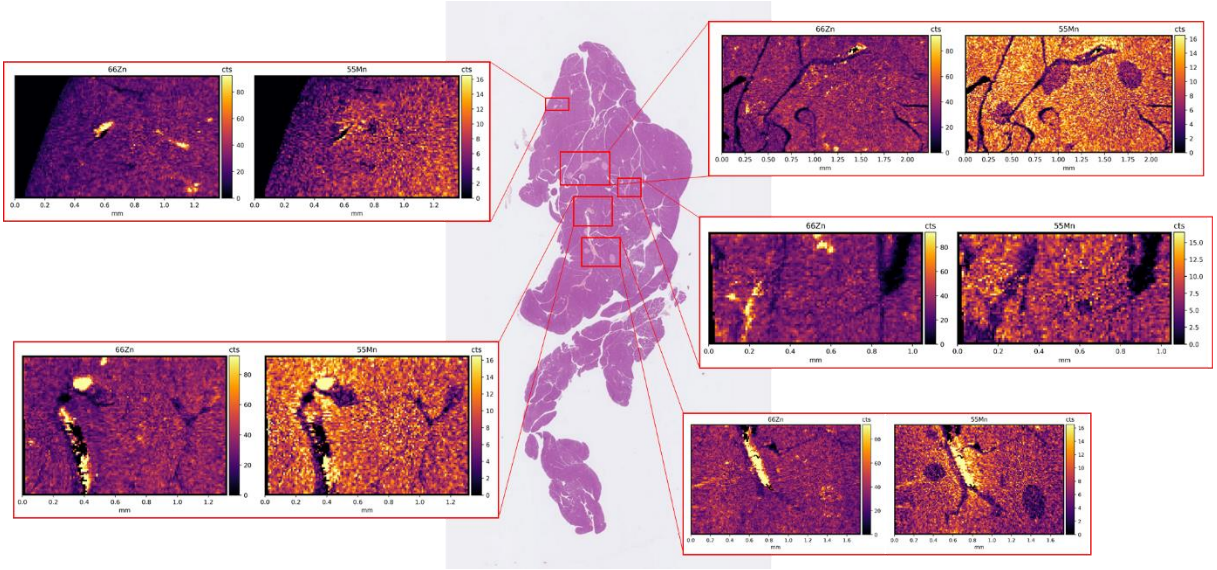

Figure S4 **LA-ICP-MS of a pancreas from a homozygous R138X mouse.** ^{66}Zn and ^{55}Mn images are shown with ablated regions highlighted on a haemotoxylin and eosin-stained pancreas to visualise exocrine and islet tissue.

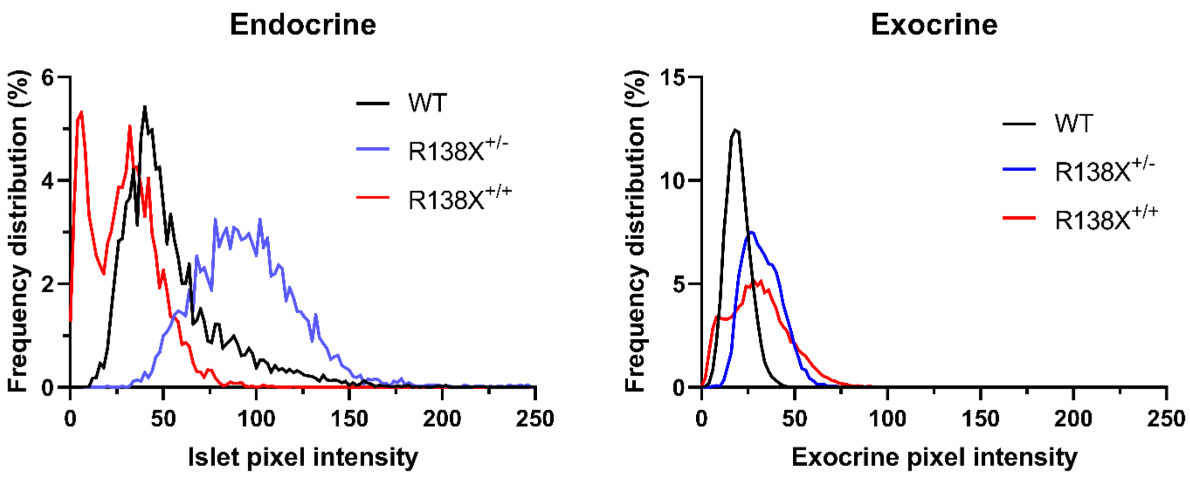

Figure S5 Zinc is heterogeneously distributed across the pancreas in R138X mice. Frequency distributions with a bin width of 2.

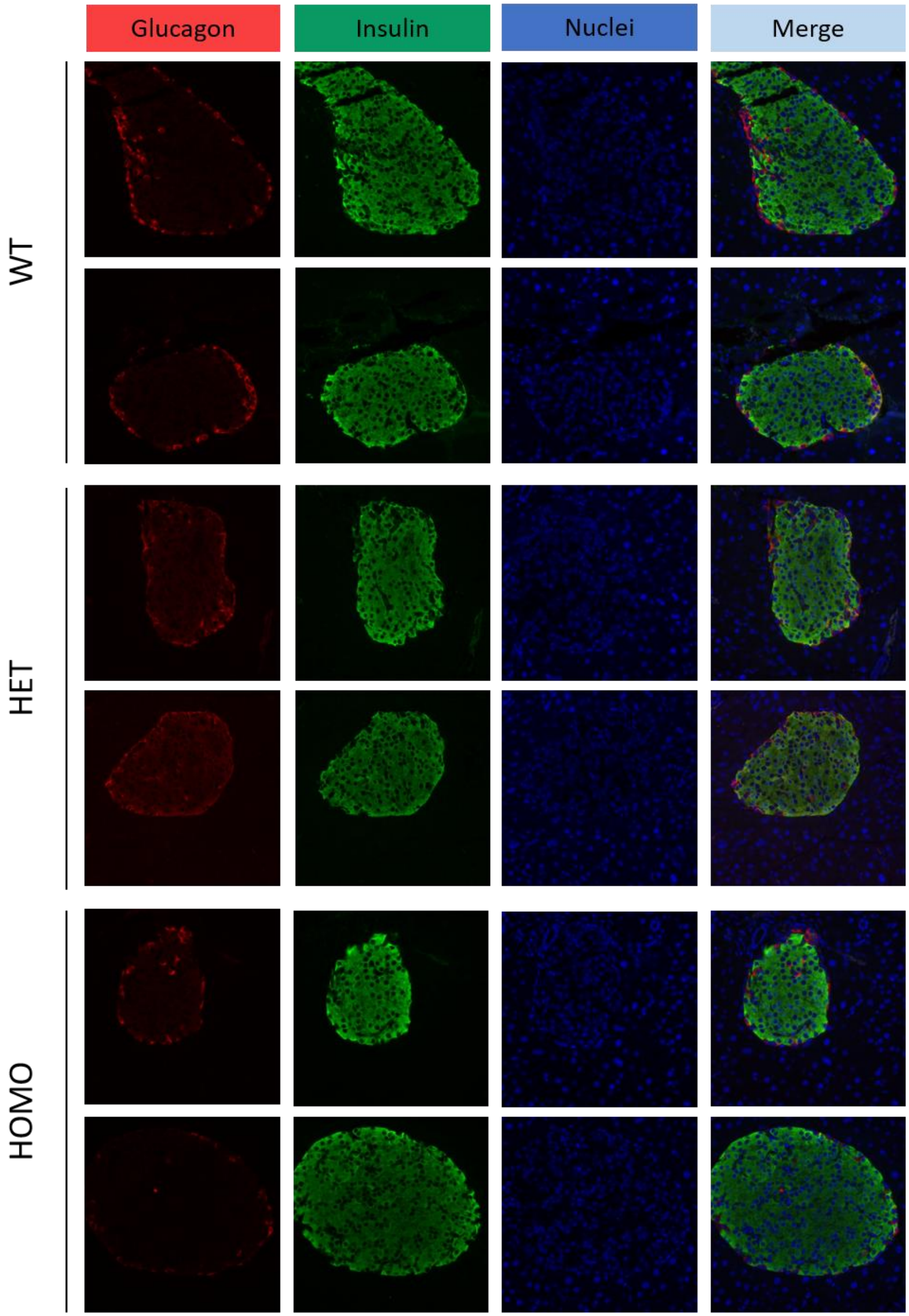

Figure S6 Histology of R138X and WT mouse islets. Histological sections (5 µm thick) of mouse pancreas were stained for islet hormones. All islets show the typical core-mantle arrangement of β and non-β cells. α cells, red; β cells, green; nuclei, blue.

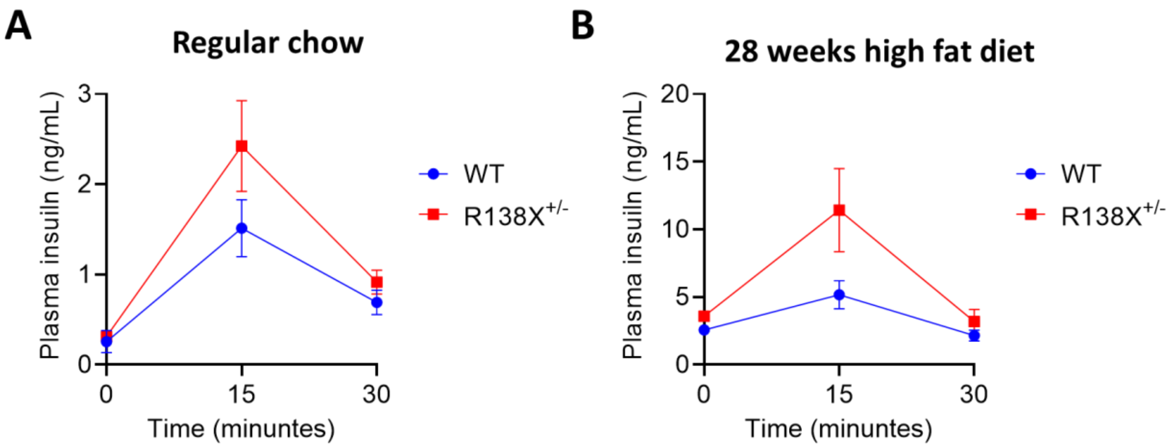

Figure S7. Glucose-stimulated insulin secretion in vivo. Insulin was measured at the indicated time points after injection of glucose (2 g/kg). Animals were maintained on regular chow (A) or on High Fat Diet (B). Other details are provided under Methods. No significant changes were detected by ANOVA.
